# Unexpected

**DOI:** 10.3201/eid1511.090279

**Published:** 2009-11

**Authors:** Veranja Liyanapathirana

**Affiliations:** University of Peradeniya, Peradeniya, Sri Lanka

He came in for a urethral job
TURP is what the doctor said
Nothing serious
He sought treatment because his son insisted
“What is it with getting up twice at night?”
“No Thaththa,^1^ it’ll be a safe surgery”
“OK Putha,^2^ you know the best”
For he was proud that his son was a nurse
The day dawned; he came to the ward,
With son and wife, and daughter to be
The harried young intern
Gave an uninterested look
“Routine admission, nothing much”
“BP normal, pulse regular, heart in dual rhythm”
“DT form filled, theatre list made”
Anaesthetist came for the premed
“Healthy gentleman, nothing to worry”
“Is there a slight murmur?”
“No, can’t be, the HO would have heard”
Surgery goes well,
Recovery uneventful,
Bladder irrigation continued uneventfully.
His son the nurse and daughter to be the nurse
Were always there anyway, most of the nurses were their friends
So things moved smoothly
Post op day 3
Fever spike
“Nothing to worry, just a UTI,
Change the antibiotic,
Send urine for culture”
The urologist said
A week went by,
Fever continued to spike
His son the nurse spoke to the urologist
“What can it be, Sir?”
“Nothing to worry, just a UTI”
“But the culture had no growth”
“He was on prophylactic antibiotics”
Day 10 came, He developed seizures,
“What can it be?”
“DVT with emboli shooting”
“Let’s do a CT”
“CT normal”
“Get a duplex of lower limb”
“Also normal”
Fever continues
Poor man, felt so unwell
Just by chance
Another intern kept the steth on his chest
“Oh my god, the murmur of MR”
“Take blood cultures”
“Do CRP”
“Get an Echo done”
On the way to the echo room, his left side goes numb
“Oscillatory vegetation in mitral valve”
“Start Pen and Gen”
Next day comes,
“MRSA isolated from blood cultures”
The dreaded report
Now what to give?
“Call the microbiologist”
“It has usually got a bad prognosis”
“Call the cardiologist”
Arguments, consultations
In the midst of it,
His son looks helpless
“Why did I push Thaththa to undergo surgery?”
The father looks on
Oblivious to the commotion
But deep within
He knows
That something’s not right
“My son’s wedding
My wife’s future”
He thinks…………

## Footnotes

1 Father.

2 Son.

